# Effect of xylazine, detomidine, medetomidine and dexmedetomidine during laparoscopic SCNT embryo transfer on pregnancy rate and some physiological variables in goats

**DOI:** 10.1186/s12917-022-03194-8

**Published:** 2022-03-15

**Authors:** Seyed Morteza Aghamiri, Amir Saeed Samimi, Mehdi Hajian, Amir Masoud Samimi, Amin Oroumieh

**Affiliations:** 1 Department of Clinical Sciences, Faculty of Veterinary Medicine, Shahid Bahonar University of Kerman, Kerman, Iran; 2 Department of Animal Biotechnology, Reproductive Biomedicine Research Center, Royan Institute for Biotechnology, ACECR, Isfahan, Iran; 3 San Diego University for integrative Studies, San Diego, USA

**Keywords:** Physiological variables, Pregnancy rate, SCNT embryo, Laparoscopy, Goats

## Abstract

**Background:**

The present study was conducted to determine if using α_2_-adrenergic agonists results in decreased stress levels (lower cortisol levels) in goats used for laparoscopic embryo [somatic cell nuclear transfer (SCNT)] transfer; and there is an effect on pregnancy rate when stress levels are lessened. Sixty healthy does aged 24 ± 4 months and weighing 30 ± 3 kg were used in experimental, prospective, randomized and blinded study. In this study, embryos were obtained by the Somatic Cell Nuclear Transfer (SCNT) method. Animals were randomly assigned to five groups: control (normal saline); xylazine (100 μg kg^− 1^); detomidine (50 μg kg^− 1^); medetomidine (20 μg kg^− 1^); and dexmedetomidine (5 μg kg^− 1^). Embryo transfer (through laparoscopic technique) began at 15 min and continued till 45 min post-treatment. Heart rate (HR), respiratory rate (RR), rectal temperature (RT), and ruminal motility were performed before (baseline) and after drug administration. Pregnancy detection was performed 38 days after embryo transfer.

**Results:**

Compared to control, HR, RR and ruminal motility were significantly lower in α_2_-adrenergic agonists groups at 5–90, 15–60, and 5–120 min, respectively. Serum cortisol values significantly increased from baseline in the control group 45 min after drug administration (*p* = 0.001). At time points 45 and 60 min, serum cortisol concentration was significantly lower in α_2_-adrenergic agonists groups compared with the control. The pregnancy rate in control group (*n* = 4/12, 33.3%) was significantly lower than xylazine (*n* = 9/12, 75%; *p* = 0.041), detomidine (*n* = 10/12, 83.3%; *p* = 0.013), medetomidine (*n* = 9/12, 75%; *p* = 0.041) and dexmedetomidine (*n* = 10/12, 83.3%; *p* = 0.013); but no significant differences were observed among different α_2_-adrenergic agonists groups.

**Conclusion:**

Alph_2_-adrenergic agonists were effective on increasing the pregnancy rate of recipient goats receiving cloned embryos. No significant differences were detected among different α_2_-adrenergic agonists.

## Background

In addition to physical restraint, chemical agents are useful and often necessary to ensure immobility and to provide sedation and analgesia for surgical and non-surgical procedures in the veterinary patients [1, 2]. Many α_2_-adrenergic agonists and narcotics are commonly used for sedation, analgesia, and anesthesia in ruminants [3–5].

Embryo transfer is an important technique to improve pregnancy rate as well as provides an opportunity to utilize the genetic contribution. Laparoscopy is one of the most useful and non-invasive surgical techniques used in small ruminants for embryo transfer [1, 6, 7] and artificial insemination [8]. Even this less invasive technique is not without stress and/or risks that may result in decreased pregnancy rates and poorer reproductive performance [9]. The premise of this study is that using sedation via α_2_-adrenergic agonists will improve outcomes.

According to the significant effects of α_2_-adrenergic agonists on cortisol level and physiological variables [10, 11] and since there is a distinct lack of documented information on the effects of α_2_-adrenergic agonists on pregnancy rate during laparoscopic embryo transfer in small ruminants, The present study was conducted to determine if using α_2_-adrenergic agonists results in decreased stress levels (lower cortisol levels) in goats used for laparoscopic embryo [somatic cell nuclear transfer (SCNT)] transfer; and there is an effect on pregnancy rate when stress levels are lessened. It is hypothesized that the effects of α_2_-adrenergic agonists during laparoscopic SCNT embryo transfer would affect the pregnancy rate after IV administration in goats.

## Results

All animals finish the study, and the procedure was not repeated in any does. Half of the goats in each group were showed vocalization (in different patterns) during the procedures. Also, two animals in each group were showed some complications (such as regurgitate, salivate, aspirate and bloat) after the procedures. All goats had recovered by 3 h based on behavioral signs such as standing, head up, head and ear movement, consciousness, and responsiveness. The animals had normal health status 48 h after the study. Physiological variables are indicated in Table 1. According to the results, HR and RR significantly increased from baseline at 15–45 min after normal saline administration. In XYL, DET, MED, and DEX, HR significantly decreased from baseline at 5–120 min after drug administration. HR was significantly lower in α_2_-adrenergic agonists groups at 5–90 min compared to the control. In α_2_-adrenergic agonists groups, a significant decrease from baseline in RR was detected between 30 and 60 min. RR was significantly lower in α_2_-adrenergic agonists groups at 15–60 min compared to the control. Ruminal motility was decreased in α_2_-adrenergic agonists groups at 5, 90, and 120 min and absent at 10–60 compared to the baseline. Compared to the control, ruminal motility was significantly lower in α_2_-adrenergic agonists groups at 5–120 min after drug administration. There were no significant differences in HR, RR, and ruminal motility among α_2_-adrenergic agonists groups at all time points. No significant differences were observed in RT at different times in each group or among groups. CRT (capillary refill time) was less than 2 sec at all time points following each drug.

**Table 1:** Comparing xylazine, detomidine, medetomidine on physiological variables (mean ± standard deviation) during laparoscopic embryo (somatic cell nuclear transfer) transferring in 60 does. Animals were assigned to five intravenous groups: control (normal saline, 5 mL); XYL, xylazine (100 μg kg^− 1^); DET, detomidine (50 μg kg^− 1^); MED, medetomidine (20 μg kg^− 1^); and DEX, dexmedetomidine (5 μg kg^− 1^)

Varaibles	Groups	Time (minutes)								
		Baseline	5	10	15	30	45	60	90	120
HR (beats minute^− 1^)	Control	107 ± 5	106 ± 5	107 ± 3	118 ± 4 ^†^	118 ± 3 ^†^	119 ± 3 ^†^	107 ± 4	108 ± 3	106 ± 4
	XYL	105 ± 6	100 ± 6 ^*†^	95 ± 5 ^*†^	90 ± 6 ^*†^	80 ± 6 ^*†^	79 ± 4 ^*†^	81 ± 4 ^*†^	87 ± 4 ^*†^	106 ± 5
	DET	108 ± 3	100 ± 2 ^*†^	97 ± 3 ^*†^	94 ± 4 ^*†^	84 ± 4 ^*†^	82 ± 4 ^*†^	84 ± 3 ^*†^	89 ± 2 ^*†^	107 ± 3
	MED	103 ± 4	98 ± 3 ^*†^	93 ± 5 ^*†^	89 ± 4 ^*†^	81 ± 3 ^*†^	79 ± 4 ^*†^	82 ± 3 ^*†^	87 ± 3 ^*†^	103 ± 3
	DEX	107 ± 6	100 ± 4 ^*†^	96 ± 6 ^*†^	92 ± 5 ^*†^	82 ± 5 ^*†^	83 ± 5 ^*†^	83 ± 4 ^*†^	87 ± 4 ^*†^	107 ± 6
RR (breaths minute^− 1^)	Control	29 ± 2	28 ± 2	28 ± 2	32 ± 1 ^†^	32 ± 1 ^†^	32 ± 1 ^†^	28 ± 1	28 ± 1	29 ± 1
	XYL	28 ± 1	27 ± 1	27 ± 1	26 ± 1 ^*†^	24 ± 1 ^*†^	23 ± 1 ^*†^	25 ± 1 ^*†^	27 ± 1	28 ± 1
	DET	28 ± 2	27 ± 2	27 ± 2	26 ± 2 ^*†^	24 ± 2 ^*†^	23 ± 2 ^*†^	25 ± 1 ^*†^	27 ± 2	28 ± 2
	MED	29 ± 2	28 ± 2	28 ± 2	27 ± 2 ^*†^	25 ± 2 ^*†^	24 ± 2 ^*†^	25 ± 2 ^*†^	27 ± 2	28 ± 2
	DEX	29 ± 2	28 ± 2	28 ± 2	27 ± 2 ^*†^	24 ± 2 ^*†^	24 ± 2 ^*†^	25 ± 2 ^*†^	27 ± 2	28 ± 1
RT (° C)	Control	38.8 ± 0.2	38.8 ± 0.2	38.8 ± 0.3	38.9 ± 0.2	38.6 ± 0.3	38.6 ± 0.2	38.6 ± 0.2	38.7 ± 0.1	38.6 ± 0.1
	XYL	38.7 ± 0.4	38.6 ± 0.1	38.7 ± 0.2	38.8 ± 0.2	38.8 ± 0.2	38.6 ± 0.2	38.7 ± 0.2	38.5 ± 0.2	38.8 ± 0.2
	DET	38.6 ± 0.3	38.7 ± 0.2	38.6 ± 0.2	38.6 ± 0.2	38.8 ± 0.2	38.8 ± 0.3	38.8 ± 0.3	37.8 ± 0.3	38.6 ± 0.2
	MED	38.8 ± 0.1	38.8 ± 0.1	38.7 ± 0.2	38.7 ± 0.2	38.6 ± 0.2	38.6 ± 0.2	37.9 ± 0.3	37.8 ± 0.3	38.7 ± 0.2
	DEX	38.6 ± 0.2	38.7 ± 0.1	38.6 ± 0.2	38.7 ± 0.2	38.7 ± 0.1	38.7 ± 0.3	37.7 ± 0.3	38.7 ± 0.2	38.6 ± 0.3
Ruminal motility (contraction minutes^− 1^)	Control	2	2	2	2	2	2	2	2	2
	XYL	2	1 ^*†^	0 ^*†^	0 ^*†^	0 ^*†^	0 ^*†^	0 ^*†^	1 ^*†^	1 ^*†^
	DET	2	1 ^*†^	0 ^*†^	0 ^*†^	0 ^*†^	0 ^*†^	0 ^*†^	1 ^*†^	1 ^*†^
	MED	2	1 ^*†^	0 ^*†^	0 ^*†^	0 ^*†^	0 ^*†^	0 ^*†^	1 ^*†^	1 ^*†^
	DEX	2	1 ^*†^	0 ^*†^	0 ^*†^	0 ^*†^	0 ^*†^	0 ^*†^	1 ^*†^	1 ^*†^
Cortisol (nmol/l)	Control	59 ± 11	–	–	–	–	80 ± 8 ^†^	–	–	61 ± 14
	XYL	60 ± 13	–	–	–	–	34 ± 6 ^*†^	–	–	48 ± 4 ^*†^
	DET	63 ± 12	–	–	–	–	34 ± 7 ^*†^	–	–	45 ± 3 ^*†^
	MED	60 ± 13	–	–	–	–	36 ± 4 ^*†^	–	–	46 ± 5 ^*†^
	DEX	63 ± 11	–	–	–	–	35 ± 5 ^*†^	–		44 ± 3 ^*†^

*HR* Heart rate, *RR* Respiratory rate, *RT* Rectal temperature

^*^Significantly different from control at the same time point (*p* < 0.05)

^†^ Significantly different from baseline value within the same group (*p* < 0.05)

Serum cortisol values significantly increased from baseline in the control group 45 min after normal saline administration (*p* = 0.001). At time points 45 and 120 min, serum cortisol concentration was significantly lower in α_2_-adrenergic agonists groups compared tho the control; however, no significant differences were observed among different α_2_-adrenergic agonists (Table 1). serum cortisol concentration decreased from baseline in α_2_-adrenergic agonists groups at 45 and 120 min after drug administration.

The pregnancy rate in control group (*n* = 4/12, 33.3%) was significantly lower than XYL (*n* = 9/12, 75%; *p* = 0.041), DET (*n* = 10/12, 83.3%; *p* = 0.013), MED (*n* = 9/12, 75%; *p* = 0.041) and DEX (*n* = 10/12, 83.3%; *p* = 0.013); but no significant differences were observed among α_2_-adrenergic agonists groups. The animals were not pregnant were not used again.

## Discussion

α_2_-adrenergic agonists are widely used in sedation, analgesia, and restraint of small ruminants. These drugs bind to α_2_-agonist receptors in the brain and spinal cord [5]. α_2_-agonist’ doses used in the present study were determined based on the doses of these drugs used in other studies in small ruminants [12–14]. With regard to anesthesia, goats respond well to standard doses of α_2_-adrenergic agonists. Medetomidine at 20 μg kg^− 1^ is recommended in sheep by Moolchand et al. (2014) [12]. The xylazine and detomidine doses used in the present study were determined based on Shah et al.’s study [14]. Dexmedetomidine at 5 μg kg^− 1^ is recommended by Seddighi and Doherty (2016) [13]. Nowadays, the laparoscopy technique is widely used in many veterinary procedures (e.g., embryo transfer) [6, 8]. Reducing stress levels in animals during veterinary interventions such as laparoscopy was emphasized [9, 15].

The goats in each group were showed some side effects (such as vocalization, regurgitation, salivation, aspiration, and bloat) during this study. Recumbency in a sedated ruminant is potentially dangerous since they can regurgitate and possibly aspirate this fluid [16, 17]. De Carvalho et al. (2016) demonstrated that IV administration of xylazine in sheep leads to drooling and urination [4]. Sheep exhibited clinical signs of mydriasis, urination, drooling, and vocalization after IV administration of dexmedetomidine IV [3]. Alph_2_-adrenergic agonists greatly affect oxygenation also [3]. With the positioning used in this study and using the insufflations of abdomen for laparoscopy, these animals are very likely to suffer hypoxemia. Therefore, the animals in this study were given oxygen to try to avoid any hypoxemia.

HR and RR significantly increased from baseline at 15–45 min, and cortisol significantly increased from baseline at 45 min in the control group. The incidence of laparoscopy-related stress and physiological changes during artificial insemination [8] and embryo transfer [1, 15, 18] was reported in ruminants. Stress and physiological changes can reduce pregnancy rate and productivity in husbandry units [6, 9, 15]. It is well known that stress increases in plasma corticoid concentrations, which are regulated by the corticotrophin-releasing factor that is secreted by the hypothalamus in response to stress. Corticotrophin-releasing factor acts at additional sites in the central nervous system to stimulate sympathetic noradrenergic outflow to the cardiorespiratory rate and inhibit cardiorespiratory parasympathetic nervous activity, resulting in increased heart and respiratory rate [9, 10].

α_2_-adrenergic agonists produced significant reduction in RR between 30 and 60 min after administration. Furthermore, these drugs a significant reduction in HR 5 to 90 min and ruminal motility 5 to 120 min after administration. The decrease in physiological variables (HR, RR, and ruminal motility) following α_2_-adrenergic agonists were reported in other small ruminants such as sheep and goats [16, 17] and camels [2]. By affecting the hormonal and nervous systems, α_2_-adrenergic agonists reduce HR, RR, and gastrointestinal motility [5, 10, 19]. The serum cortisol levels were significantly lower in different α_2_-adrenergic agonists groups than in normal saline. Reduction in the concentration of serum cortisol level following α_2_-adrenergic agonists has been reported in previous studies with another stress such as surgery or restraint or even isolation from other animals [9, 10]. α_2_-adrenergic agonists inhibited cortisol secretion by a mechanism involving α_2_-adrenergic receptors or via direct suppression of adrenocorticotropic hormone (ACTH) release at the pituitary level [20].

The pregnancy rate of embryos (SCNT) transferred was significantly higher in different α_2_-adrenergic agonists groups than in normal saline. Paramio and Izquierdo (2014) have stated the average of SCNT overall efficiency (live kids/embryo transferred) was low [21]. The pregnancy rate of SCNT embryos in Baguisi et al. (1999)’ study was lower than this study. It may be due to the use of sedative drugs in this study [22]. The use of sedatives during laparoscopic examination in sheep [9] and laparoscopic embryo transfer in sheep and goats [18] was recommended. Using α_2_-adrenergic agonists results in decreased stress levels (lower cortisol levels) in goats used for laparoscopic embryo (SCNT) transfer; and there is an effect on pregnancy rate. Laparoscopic technique and restraint steps were considered as two major stressors in the animals of the present study. In this study, only two embryos were transferred into uterine horns of recipient goats that was lesser comparing other studies using uterine horns [23] or tubal [24] embryo transfer. Moreover, similar to this experiment, other studies have been shown just one kid was born from every recipient in most cases [7, 23, 24].

SCNT could be a valuable technique for genetic improvement in goats. Furthermore, providing recombinant proteins are possible by transgenic goats produced via SCNT method [21, 23]. However, SCNT efficiency is low [21] and many oocytes are required to produce a few number of SCNT blastocysts. Therefore, the SCNT embryos are valuable and relatively expensive. Every method helps to achieve good pregnancy rate by fewer of them is worth. Fonseca et al. (2016) suggested that the improved technique, better animal welfare, and shorter surgery time could be helpful for embryo transfer [18]. There are articles using other procedures (electroejaculator, rumenotomy, etc.) that suggest the use of anesthetics/sedatives improves the outcome they are looking for [19]. Good restraint is important to getting a producer done efficiently and correctly. Actual immobility and lack of response to surgery by the patient likely helps the surgeon perform the embryo transfer properly and more quickly. Most all surgeons can do a better job when the animal is not moving, struggling, and/or vocalizing [13].

The examined animals were laid upside down from 5 to 45 min after drug administration; therefore, the assessed duration and quality of sedation might be erroneous. More frequent measurement of cortisol to see if it is increased during surgery or was low throught, would have been very useful. Another limitation was regarding about extravascular injection during administration of the drugs. Althought all injection were recorded as being smooth with no obvious extravasation of the drug but it is hard to be sure when not giving through a catheter. Measurement of partial pressures of oxygen and carbon dioxide, arterial blood pressure, and pH would describe the effects of α_2_-adrenergic agonists during during laparoscopic SCNT embryo transfer.

## Conclusion

Alph_2_-adrenergic agonists were effective on increasing the pregnancy rate of recipient goats receiving cloned embryos. No significant differences were detected among xylazine (at 100 μg kg^− 1^), detomidine (at 50 μg kg^− 1^), medetomidine (at 20 μg kg^− 1^) and dexmedetomidine (at 5 μg kg^− 1^). More investigations with more frequent evaluation of cortisol and longer periods of monitoring (at least until parturition) are recommended.

## Methods

### Animals

The study was approved by the animal welfare commission of the Faculty of Veterinary Medicine, Shahid Bahonar University of Kerman (no. IR.UK.VETMED.REC.1399.009). Sixty healthy does aged 24 ± 4 months and weighing 30 ± 3 kg [mean ± standard deviation (SD)] were used. The animals were selected from the Animal Husbandry Unit of Royan Institute for Biotechnology, Isfahan (latitude 32°67*′*N and longitude 51°77′E), Iran using a sample lottery method (simple randomization). All animals were housed under the same husbandry, nutritional, and management conditions in the same group pen. The animals received a constant mixture containing roughages (mainly alfalfa hay and wheat straw) and concentrate (barley grain, soybean meal, wheat bran) based on physiological maintenance during the experiment. The forage/concentrate ratios during the experiment were 90:10. Minerals and vitamins were also added to the rations. Fresh water was also provided. Two months before the experiment, animals were treated with broad-spectrum antiparasitic drugs for probable internal and external parasitic infestation. The health status of all animals was checked routinely by clinical (including heart and respiratory rate, rectal temperature, capillary refill time, and ruminal motility) and paraclinical examinations. The paraclinical examination consisted of hematological (evaluation of complete blood count and packed cell volume) and fecal parasitic analysis. The does had no history of reproductive problems, and they were bred successfully in the past. Any of does didn’t have difficulty becoming pregnant.

The study was performed during the breeding season. Before the experiment, food and water were withheld from the goats for 12 and 6 h, respectively. The experiment was carried out in the morning. The animals were weighed for the calculation of drug dosages. One animal was studied at any one time. The animals were unable to see or interact with each other. The skin over the left jugular vein was prepared aseptically for IV administration and blood sampling. Animals rested for 20 min before drug administration (baseline). All injections were recorded as being smooth with no obvious extravasation of the drug. The person who injected each time was the same.

### Experimental procedures

**Fig. 1:**
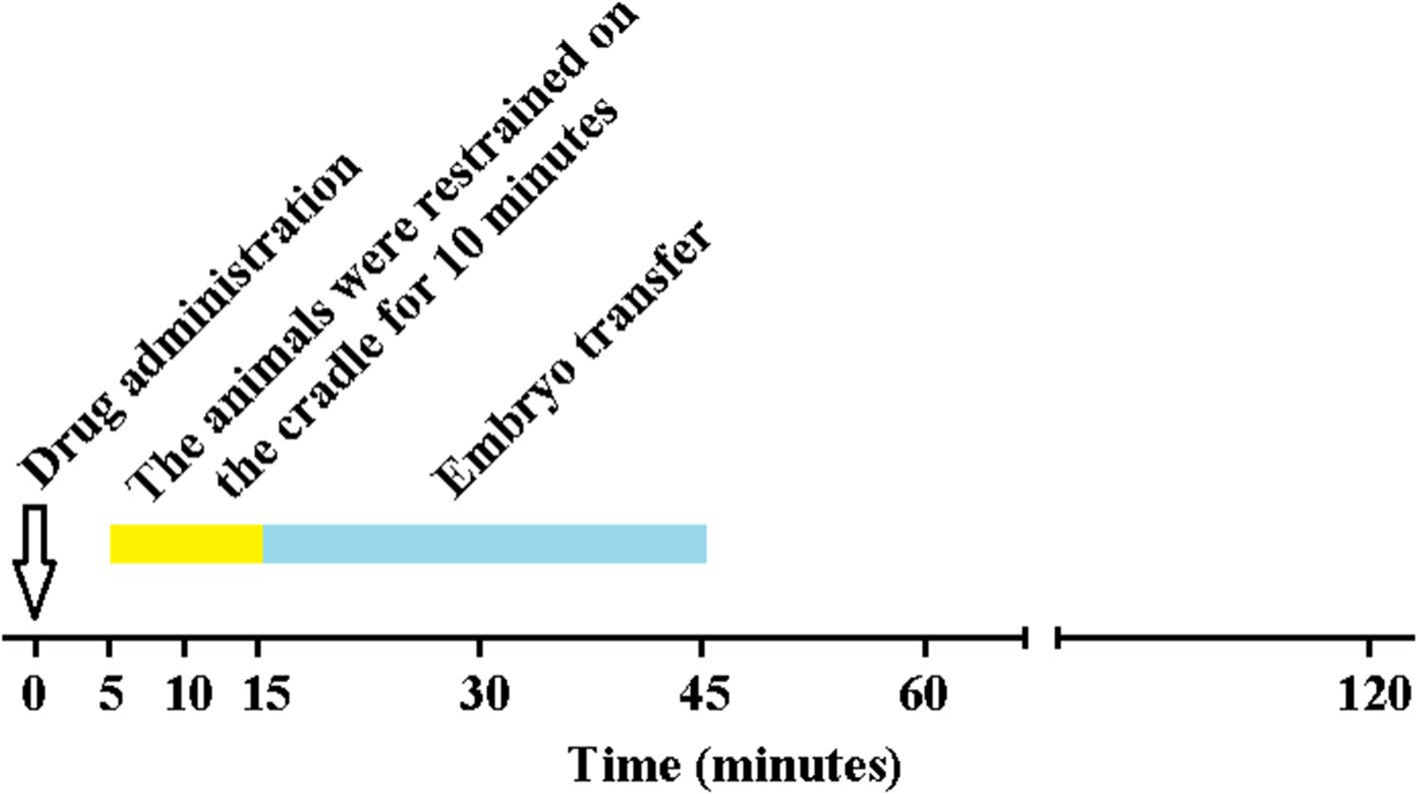
Schematic diagram of present experimental design. Animals rested for 20 minutes before drug administration (baseline). Drugs were administered intravenously in the left jugular vein with the animals standing. Animals were placed on the special cradle 5 minutes after the treatment. The animals were gently restrained for laparoscopy and placed on the special 45° cradle on top of a soft mattress for 10 minutes. Embryo transfer began at 15 minutes and continued till 45 minutes post-treatment. The detection of pregnancy in animals was performed by transabdominal B-mode ultrasonography 38 days after embryo transfer

Animals were assigned randomly to five IV groups: control (normal saline, 5 mL); XYL, xylazine (100 μg kg^− 1^; Xyla, 2%; Interchemie Werken De Adelaar B.V., Netherlands); DET, detomidine (50 μg kg^− 1^; Domosedan, Orion Corporation, Finland); MED, medetomidine (20 μg kg^− 1^; DorbeneVet; N-Vet AB, Sweden); and DEX, dexmedetomidine (5 μg kg^− 1^; Dexdomitor; Orion Corporation, Finland). The injection volumes of treatments were the same for each animal by dilution with normal saline to 5 mL. Drugs were administered IV in the left jugular vein (over 20 s) via an 18 gauge needle with the animals standing. Animals were placed on the special cradle 5 min after the treatment (Fig. 1). The embryos were produced via SCNT technique, according to Hajian et al. (2020)‘s study in Royan Institute for Biotechnology, Isfahan, Iran [7]. Briefly, ovaries were prepared from Fasaran abattoir, Isfahan province, Iran. Following dissection, the ovaries were maintained at 15–17 °C in normal saline with antibiotics (200 IU/mL Penicillin G and 0.2 mg/mL Streptomycin) and transferred to the laboratory. The cumulus oocyte complex (COCs) were obtained from follicular fluid, cultured in maturation medium and incubated at 38.5 °C for 20 h with 5% CO2. Then, the cumulus cells were denudated by hyaluronidase, zonae pellucidae were removed using pronase enzyme, and oocytes were enucleated by mouth pipetting. Subsequently, single fibroblast cells were fused to the enucleated oocytes membrane using a sinusoidal electric current (1.7 kV/cm). After activating by ionomycin and dimethylaminopurine, reconstructed oocytes were cultured in synthetic oviductal fluid (SOF) for 7 days at 38.5 °C. Since blastocysts were examined and only grade 1 and 2 blastocysts were chosen for embryo transfer [7]. Recipient goats were synchronized using a vaginal progesterone device (Eazi-Breed CIDR, Zoetis, Australia) for 7 days. Then, equine chorionic gonadotropin (eCG) hormone (GONASER®, HIPRA, Spain) 400 IU at day 5, cloprostenol sodium (estroPLAN; Parnell Technologies, Australia) 250 μg at day 7, and human chorionic gonadotrophin (hCG) hormone (Choragon, Ferring, Germany) 1000 IU at day 9 were administered. On day 16, 7-day embryos were transferred by laparoscopic methods recipients. The animals were gently restrained for laparoscopy embryo transfer and placed on the special 45° cradle on top of a soft mattress for 10 min. The animals laid in dorsal recumbency position with their head down in lateral on the left side, and their legs were tied. The heads were positioned so the saliva and any reflux from the gastrointestinal tract would drain away from the throat. Animals were given oxygen. The skin over the areas for the laparoscopic port (on the ventral side of the abdomen) was prepared aseptically. Local anesthetic was performed by lidocaine (0.22 mg kg^− 1^; Lidocaine 0.2%, Pasture Institute, Iran). After disinfecting the surgical area, a 10-mm trocar was used to pass telescope 0° (Hopkins II, Karl Storz, Germany) into the abdominal cavity, about 6-cm cranial to the teats and 3-cm from the midline right side away from the rumen. Ovaries were examined to detect the corpus luteum. Embryo transfer began at 15 min and continued till 45 min post-treatment. Two embryos were inserted into the equivalent uterine horn to the ovary with an active corpus luteum using a handmade pipette [1, 7, 18]. At 45 min point, animal legs were untied, and they were gently placed on the floor (with soft mattress) in sternal position and allowed to recover. The animals were positioned to try to avoid any complications such as regurgitation, aspiration, and bloat. The animals were monitored in recovery every 15 min after the procedure. All investigators recording measurements were unaware of the treatment assigned.

### Physiological variables

Physiological variables including heart rate (HR), respiratory rate (RR), rectal temperature (RT), capillary refill time (CRT), and ruminal motility were recorded at baseline (before drug administration) and 5, 10, 15, 30, 45, 60, 90, and 120 min after drug administration. HR and RR were assessed using a veterinary stethoscope (Classic SE Littmann; 3 M, MN, USA) on the left 4th and 6th intercostal space for 1 min. Ruminal motility was recorded by auscultation with a stethoscope placed on the left flank. The number of audible rumen contractions within 2 min was counted. CRT was measured by finger pressing on the labial surface of the gingiva in the incisor region. A digital thermometer (FT09; Beurer GmbH, Germany) was used to performed RT. The thermometer was 4–5 cm deep in touch with rectal mucosa for at least 2 min. Serum cortisol concentration was investigated via the radioimmunoassay method (Orion Diagnostica, Finland) at baseline, 45 and 120 min after drug administration. 45 min sample was at the end of the laparoscopy, and the goats were still restrained in the cradle. The pregnancy detection in animals was performed by transabdominal B-mode ultrasonography (Imago, ECM, France) 38 days after embryo transfer. The pregnancy rate was calculated using the following formula: Pregnancy rate = (animals pregnant/animals which had embryos transferred) × 100.

### Statistical analysis

Physiological variables (HR, RR, RT, CRT, and ruminal motility) were expressed as mean ± standard deviation (SD). Data were analyzed using SPSS software version 23.0 (SPSS for Windows, SPSS Inc., Chicago, Illinois). Befor any statistical analysis, distribution of data was performed for normality using the Kolmogorov-Smirnov test and normality of data distribution was verified. One-way analysis of variance (ANOVA) with Tukey’s post hoc test was used to compare mean values of physiological variables at similar times between different groups. The repeated measures ANOVA in general linear model was applied to compare physiological variables at different times from baseline in each group. In this model, animals in each group were included as repeated subject. Moreover, nine time points (to evaluate physiological variables) were considered as nine levels, respectively. Also, three time points (to measurement cortisol level) were considered as three levels, respectively. A chi-square test was performed to compare the rates of pregnancy among different groups. Differences were considered statistically significant when the calculated *p*-value was less than 0.05.

## Data Availability

All data generated or analyzed during this study are available from the corresponding authors on reasonable request.

## Abbreviations

SCNT: Somatic cell nuclear transfer
HR: Heart rate
RR: Respiratory rate
RT: Rectal temperature
IV: Intravenous
CRT: Capillary refill time
XYL: Xylazine
DET: Detomidine
MED: Medetomidine
DEX: Dexmedetomidine
eCG: Equine chorionic gonadotrophin
SD: Standard deviation.
